# Seed priming with lavender essential oil increases germination and seedling growth in saline and potentially toxic elements-contaminated soils

**DOI:** 10.7717/peerj.20717

**Published:** 2026-04-08

**Authors:** Giovanni Kamal Piga, Marco Cossu, Vittoria Giannini, Laura Frabboni, Grazia Disciglio, Annalisa Tarantino, Onofrio Marco Pistillo, Angela Racioppo, Matteo Garau, Giovanni Garau, Sara Melito

**Affiliations:** 1 Department of Agricultural Sciences, University of Sassari, Sassari, Sardinia, Italy; 2 Innovative Agriculture Interdepartmental Center, Alghero, Sardinia, Italy; 3 Department of Agronomy, Food, Natural Resources, Animals and Environment, University of Padua, Legnaro, Italy; 4 Department of Land, Environment, Agriculture and Forestry, University of Padua, Legnaro, Italy; 5 Department of Agricultural, Food and Environmental Sciences, University of Foggia, Foggia, Italy

**Keywords:** Pollution, Heavy metals, Soil tolerance, Aromatic plants, Marginal areas, Abiotic stress

## Abstract

**Background:**

Seed priming with natural compounds has emerged as a promising strategy to enhance plant tolerance to abiotic stresses. Among these, essential oils may mitigate the adverse effects of potentially toxic elements (PTEs) and salinity on seed germination and early growth. This study aimed to investigate the priming effects of *Lavandula angustifolia* Mill. essential oil on germination and seedling development of 3 Mediterranean aromatic species: *Achillea millefolium* L., *Ocimum basilicum* L., and *Thymus vulgaris* L.

**Methods:**

Priming treatments were performed using different essential oil concentration (0.1%, 0.2%, 0.4%, 0.8%) with H_2_O and polysorbate P80 controls. Seed were grown on two heavy metal-polluted soils collected from abandoned mining sites (Montevecchio, HM1; Su Suergiu, HM2; Sardinia, Italy), a saline soil from Arborea in Sardinia (Sal) and an unpolluted sandy control soil. HM1 and HM2 soils were contaminated with arsenic (As), cadmium (Cd), lead (Pb), antimony (Sb) and zinc (Zn).

**Results:**

Soil type, plant species, and priming conditions significantly affected germination rate, seedling vigor index, and fresh weight. Notably, 0.2% essential oil priming did not markedly enhance germination compared to controls, except for *A. millefolium* grown on the contaminated HM1 soil and *T. vulgaris* on control soil. Both *A. millefolium* and *T. vulgaris* showed significant higher soil tolerance indices in contaminated soils under 0.2% priming, while *T. vulgaris* also exhibited further improvement under 0.2–0.8% priming in control soil. Moreover, in Sal soil, germination and seedling development were generally inhibited across all species; however, *L. angustifolia* essential oil at 0.2% occasionally alleviated these negative effects.

**Conclusions:**

Low-dose *Lavandula angustifolia* essential oil priming can improve germination and stress tolerance in certain aromatic species, representing a sustainable approach for the valorization of marginal and degraded lands.

## Introduction

Land marginality, affecting wide portions of world land, could be related to different causes, among which soil salinity and contamination by potentially toxic elements (PTEs) are the most significant (Mali et al., 2024). Soil salinization impacts more than 8.0% of global land while approximatly 14–17% of cropland is affected by heavy metals pollutions (Hou et al., 2025), with severe consequences for food production and human health (Roy et al., 2022; FAO, 2021). In the European Union 6.24% of agricultural land requires remediation due to pollution by PTEs such as arsenic (As), cadmium (Cd), chromium (Cr), copper (Cu), mercury (Hg), lead (Pb) and zinc (Zn) (Tóth et al., 2016).

To prevent further abandonment and underuse of marginal lands, aromatic plants can be cultivated on soils affected by salinity and PTEs contamination, thereby restoring productivity and economic value to these areas. Globally, more than 60,000 plant species are recognized for medicinal and aromatic uses, and several thousand are commercially exploited for essential oil (EO) production. Most belong to the *Lamiaceae*, *Asteraceae*, and *Apiaceae* families, which include many species naturally adapted to arid, saline, nutrient-poor, and even heavy-metal-contaminated environments (Schippmann, Leaman & Cunningham, 2006; FAO, 2019; Gupta et al., 2021; Pandey, Verma & Singh, 2019). This ecological versatility is closely linked to their metabolic specialization: medicinal and aromatic plants produce a wide array of EOs and bioactive compounds, and their adaptive success under stress is often associated with complex physiological and biochemical mechanisms—such as osmolyte accumulation, enhanced antioxidant activity, and modifications of root system architecture—that sustain growth and metabolic efficiency under adverse conditions (Figueiredo & Barroso, 2015; Yadav, Verma & Singh, 2023).

Indeed, previous studies have shown that aromatic plants can grow in degraded soils, while simultaneously improving soils structure, enhancing beneficial microbes activity and stabilizing the land thereby increasing its productivity and long term sustainable (Aishwath & Lal, 2016; Pradhan et al., 2017; Poschenrieder et al., 2012; Licata et al., 2022; Gupta et al., 2021). Moreover, their biomass can be used for EO extraction and the residual by-products can be valorized for bioenergy production (*e.g.*, biogas) (Jisha, Bauddh & Shukla, 2017). From this perspective, cultivating aromatic plants on marginal soils offers a dual opportunity: it enables the recovery of otherwise unproductive lands and provides high-value products such as EOs.

Globally, the EOs world market was valuated around 7.51 billion USD in 2018 and is projected to reach 15 billion USD by 2026 with a 9% compound annual growth rate between 2019 and 2026 (Ahuja & Singh, 2019). EOs are recognized as safe and possess antimicrobial, antifungal, and antioxidant properties, making them valuable in medical, cosmetic, food, and agricultural sectors (Abu-Darwish & Abu-Dieyeh, 2009; Kumar, Malik & Atri, 2021; Vora et al., 2024; Aljaafari et al., 2021). Beyond their commercial importance, their rich composition in bioactive compounds has encouraged their agricultural use as sustainable natural inputs. Specifically, EOs are employed as natural pesticides, fungicides and herbicides (Xie et al., 2017; Tanhaeian, Sekhavati & Moghaddam, 2020; Giannini et al., 2022). Interestingly, beyond their protective roles, few studies have reported biostimulant effects of EO sand plant extracts on seed germination and early seedling development (Ben-Jabeur et al., 2019; Kisiriko et al., 2021; Souri & Bakhtiarizade, 2019). These beneficial effects are largely attributed to the bioactive compounds of EOs, such as terpenes, sesquiterpenes, and oxygenated molecules, which can enhance nutrient uptake, biomass accumulation, and oxidative stress mitigation (Licata et al., 2022; Ben Saad et al., 2024; Bakkali et al., 2008; Souri & Bakhtiarizade, 2019). Such bioactivity has led to growing interest in using EO to improve germination and seedling vigor, even under abiotic stress (Ella, Dionisio-Sese & Ismail, 2011). In this context, EOs from rosemary, eucalyptus, thyme and tansy have shown particularly promising results, supporting plant development and stress tolerance (Souri & Bakhtiarizade, 2019; Chrysargyris et al., 2020; Ben-Jabeur et al., 2019; Beni et al., 2020); more recently they have been also investigated as potential seed-priming agents (Soudani et al., 2022; Oğuz, Oğuz & Güler, 2023).

Seed priming is a pre-sowing treatment that hydrates seeds to trigger metabolic processes necessary for germination, without allowing radicle emergence, enhancing seed vigor and stress tolerance under abiotic stress conditions, particularly salinity and PTEs (Paparella et al., 2015; Khan et al., 2022; Prajapati, Kataria & Jain, 2020; Jisha, Vijayakumari & Puthur, 2013). Recently, the use of EOs as seed priming agents has emerged as an innovative strategy, showing promising effects in reducing agricultural inputs and improving plant performance under saline conditions, especially in edible crops (Devika et al., 2021; Ben Saad et al., 2024; Kesraoui et al., 2022; Oğuz, Oğuz & Güler, 2023). Although only few studies have investigated EO-based priming in aromatic plant seeds, reporting positive effects on germination and early seedling growth (Terzić et al., 2023), this approach remains scarcely investigated.

To the best of our knowledge, no previous studies have examined the effects of EO-based seed priming on aromatic plant species grown in saline and PTEs-contaminated soils. Addressing this gap is relevant for two main reasons: (i) to explore how EO seed priming influences different aromatic plant species under extreme conditions, thereby contributing to a broader understanding of its biostimulant mechanisms and applications; (ii) to enhance the establishment of aromatic plants on marginal lands which are often underutilized for food/feed crop cultivation. In saline soils, this may support EO production with high economic value (Dagar, 2018), while in metal-contaminated soils, it could enable phytoremediation efforts to produce of metal-free EOs (Angelova et al., 2015).

The present study aims to investigate the priming effect of *Lavandula angustifolia* Mill. EO on the seeds of three Mediterranean aromatic plants (*Achillea millefolium* L., *Ocimum basilicum* L. and *Thymus vulgaris* L.), which are potentially suitable for cultivation on marginal lands affected by salinity and PTEs contamination. These species exhibit varying degree of tolerance (low, medium or high) to salinity and PTEs stress (Niu & Rodriguez, 2006; Angelova & Ihtyarova, 2023) and are well known for their commercial applications in the pharmaceutical, cosmetic, and food industries. The experiments were conducted under controlled laboratory conditions and represent the first step toward developing future application under open field conditions.

## Materials & Methods

### Essential oil extraction and characterization

Lavender (*Lavandula angustifolia* Mill.) plants were transplanted in 2022 and cultivated within a dynamic agrivoltaic system (M2 Energia Srl company field) located in the marginal rural area of San Severo (Foggia, Italy; 41°4′58″N, 15°8′33″E). This system features photovoltaic solar panels positioned 2.50 m above the crops. An electronic unit controls the solar-tracking mechanism, ensuring that the panels consistently align with the sun while preventing mutual shading. The system is designed to optimize and enhance light accessibility for the underlying crops. Throughout the growing season, drip irrigation and standard agronomic practices were applied. During the balsamic phase, the lavender plants were manually harvested. The flowers were then homogenized and used for EO extraction. The freshly collected flowers of Lavender were used for hydrodistillation using a Clevenger-type apparatus (Clevenger, 1928) following the European Pharmacopoeia (Council of Europe, 2023).

Lavender essential oil (LEO) composition was determined through Gas Chromatography-Mass Spectrometry (GC-MS) (Table 1). In particular, one µl of EO (Diluted samples 1:100 (v/v) in Hexane) was analysed by an Agilent 7890B series gas chromatograph (Agilent Technologies) coupled with an Agilent 5977A mass selective detector (MSD) and equipped with a HP-5MS capillary column (30 m × 0.25 mm ID, 0.5 µm film thickness, J&W Scientific Inc., Folsom, CA, USA). The carrier gas was helium at a flow rate of 1.25 mL/min. The injection was made in splitless mode, and the injector temperature was 250 °C. The column oven temperature was programmed from 60 °C to 250 °C at 3 °C/min, with a final holding time of 10 min. Spectra were recorded in the electron impact mode (ionization energy, 70 eV) in a range of 15–550 amu at 2.9 scans/s. The identification of volatile compounds was achieved by comparing mass spectra with those of the data system library (NIST11, *p* > 90%) and wherever possible by comparing retention times and mass spectra with those of commercially available standards. Moreover, a mixture of a continuous series of straight-chain hydrocarbons, C7-C30 (Alkane Standard Solution; Sigma Aldrich, Milan, Italy) was injected under the same conditions previously described to obtain the linear retention indices (RIs) (Van Den Dool & Kratz, 1963). Component relative percentages were calculated based on GC peak areas.

**Table 1:** Chemical composition of *L. angustifolia* essential oil as identified by GC-MS.

Peak No.	Compound	R.T.	RI_Lit_^2^	RI_Exp_^3^	Area (%) ± S.D.^1^
1	α-Pinene	12.276	939	934	0.22 ± 0.01
2	Camphene	12.933	953	951	0.14 ± 0.01
3	β-Pinene	14.202	980	976	0.34 ± 0.02
4	3-Octanone	14.545	985	983	0.52 ± 0.03
5	β-Myrcene	14.741	992	989	1.49 ± 0.07
6	*p*-Cymene	16.375	1,021	1,019	0.63 ± 0.03
7	Limonene	16.582	1,029	1,031	2.34 ± 0.12
8	Eucalyptol	16.718	1,031	1,032	1.58 ± 0.08
9	cis-β-Ocimene	16.923	1,038	1,036	0.35 ± 0.02
10	trans-β-Ocimene	17.425	1,054	1,051	0.64 ± 0.03
11	Linalool oxide II (pyranoid)	18.656	1,072	1,071	0.58 ± 0.03
12	Linalool	20.127	1,099	1,097	36.81 ± 1.84
13	1-Octen-3-ol, acetate	20.430	1,112	1,111	0.10 ± 0.01
14	6,8-Epoxy-menth-1-ene	21.124	1,114	1,121	0.23 ± 0.01
15	Terpinen-1-ol	21.533	1,144	1,141	1.91 ± 0.10
16	Camphor	22.097	1,160	1,158	1.71 ± 0.09
17	endo-Borneol	23.073	1,172	1,169	0.10 ± 0.01
18	Terpinen-4-ol	23.574	1,177	1,175	0.51 ± 0.03
19	α-Terpineol	24.180	1,182	1,185	1.46 ± 0.07
20	Geraniol	25.830	1,240	1,231	0.13 ± 0.01
21	Linalyl acetate	27.205	1,247	1,251	41.01 ± 2.05
22	Geranial	27.686	1,268	1,274	0.10 ± 0.01
23	Bornyl acetate	28.432	1,287	1,285	0.56 ± 0.03
24	Lavandulyl acetate	28.512	1,292	1,291	0.52 ± 0.03
25	Neryl acetate	31.636	1,361	1,365	0.63 ± 0.03
26	α-Copaene	32.293	1,372	1,376	0.10 ± 0.01
27	Geranyl acetate	32.436	1,381	1,384	1.11 ± 0.06
28	β-Caryophyllene	34.121	1,414	1,411	0.31 ± 0.02
29	Germacrene D	37.787	1,575	1,571	0.10 ± 0.01
30	Caryophyllene oxide	40.500	1,583	1,581	0.98 ± 0.05
31	Humulene-1,2-epoxide	41.434	1,602	1,606	0.10 ± 0.01
32	α-Bisabolol	43.956	1,685	1,682	0.10 ± 0.01

**Notes.**

1 N = 3 replicates.

2 RI Lit = Linear retention index from literature.

3 RI Exp = Determined linear retention index against mixture of n-alkanes (C5–C40) on HP-5MS column.

### Soil sampling and analysis

Soil samples were collected from three different locations in Sardinia (Italy) and the soil was analysied for physico-chemical characteristics: the abandoned mining sites of Montevecchio (HM1, 39°′3′36.3″N, 8°′5′29.1″E), Su Suergiu (HM2, 39°′9′53.7″N, 9°′2′35.2″E) and the intensive agricultural area of Arborea (Sal, 39°5′23.5″N, 8°34′45.9″E). HM1 and HM2 were sampled in areas known to be polluted with PTEs (*e.g.*, Zn, Pb, antimony (Sb); Garau et al., 2023; Garau et al., 2024), whereas Sal was a saline soil with electrical conductivity (EC) of 4.99 dS m^−2^ (Table 2). Topsoil samples (*n* = 10, upper 20 cm) were collected randomly on each site and pooled together, air-dried, and sieved to less than two mm for physical-chemical characterization; pH and EC were measured in 1:5 (w/v) solid-to-water suspensions (ISO 10390:2021). Total carbon and nitrogen were measured using a CHN analyzer (Leco CHN628) with Soil LCRM Leco part number 502–697 as the calibration sample. Dissolved organic carbon (DOC) was quantified in the filtered liquid phase of a 1:10 (w/v) soil/water suspension after 24 h of agitation (Silvetti et al., 2014). Cation exchange capacity (CEC), and the concentration of exchangeable bases were determined using the BaCl2-triethanolamine method (ISO 11260:2018). The available phosphorus was quantified following Olsen method (ISO 11263:1994). The pseudo-total PTEs concentration in soils was determined by an Inductively Coupled Plasma Optical Emission Spectrometry (Perkin Elmer Optima 7300 DV ICP-OES) after microwave-assisted digestion using the US EPA Method 3051A.

**Table 2:** Physico-chemical characterization of Montevecchio (HM1) Su suergiu (HM2) and Arborea (Sal) soils.

Parameters	HM1	HM2	Sal
Texture	Sandy-clay	Sandy	Sandy
pH	6.01 ± 0.04	7.31 ± 0.06	6.9
Electrical Conductivity (dS m^−1^)	0.39 ± 0.03	2.23 ± 0.06	4.99
Soil Organic Matter (%)	3.60 ± 0.11	6.31 ± 0.02	10
Carbon/Nitrogen ratio	13.06	12.62	6
Total Organic Carbon (%)	2.09 ± 0.06	3.66 ± 0.01	6
Total Nitrogen (%)	0.16 ± 0.01	0.29 ± 0.01	1
Dissolved Organic Carbon (mg g^−1^)	0.23 ± 0.02	3.49 ± 0.15	0.35 ± 0.01
Phosphorus Available (mg kg^−1^)	22.25 ± 0.04	7.23 ± 0.67	306
Cation Exchange Capacity (cmol(+) kg^−1^)	22.78 ± 0.10	16.72 ± 0.02	11
Excangeable Na (cmol(+) kg^−1^)	0.48 ± 0.08	0.97 ± 0.22	0.05
Excangeable K (cmol(+) kg^−1^)	1.39 ± 0.05	1.72 ± 0.02	1.18
Excangeable Ca (cmol(+) kg^−1^)	19.55 ± 0.67	14.53 ± 0.95	3.37
Excangeable Mg (cmol(+) kg^−1^)	1.15 ± 0.01	2.25 ± 0.18	6.38
As (mg kg^−1^)	27.85 ± 0.23	343.15 ± 17.85	n.d
Cd (mg kg^−1^)	28.62 ± 0.10	11.91 ± 1.33	n.d.
Pb (mg kg^−1^)	10,942 ± 432.00	402.96 ± 6.34	8.86 ± 0.52
Sb (mg kg^−1^)	62.14 ± 5.15	2174 ± 122.48	n.d.
Zn (mg kg^−1^)	2,853 ± 7.00	3,148 ± 177.00	48.40 ± 0.46

### Experimental set up

A germination pot experiment was conducted to evaluate the effect of seed priming with different concentrations of LEO in different soils. The experimental design was factorial, combining four soils and six priming treatments for a total of 24 treatment combinations and three replicates as follows:

Priming treatments:

• H_2_O: distilled water (Control)
• P80: 0.18 mL of polysorbate 80 in 10 mL distilled water (Control)
• EO1: 0.1% LEO
• EO2: 0.2% LEO
• EO4: 0.4% LEO
• EO8: 0.8% LEO

Soils:

• HM1: PTEs-contaminated soil from Montevecchio
• HM2: PTEs -contaminated soil from Su Suergiu
• Sal: saline soil from Arborea
• C: control substrate (sand)

P80 was used as an emulsifier at 15% (v/v) of the EO dose to solubilize it in distilled water. EO4 was used as a stock solution to prepare the other treatment dilutions. The priming treatment was performed by imbibing approximately 150 seeds in 10 mL of solution for 2 h, followed by washing with sterile water. This duration was selected based on preliminary tests conducted for all species, which showed complete seed imbibition within 2 h without visible signs of overhydration. The preparation of these experimental solutions is detailed in Table S1.

Three aromatic species were selected for the experiment: *A. millefolium* L., *O. basilicum* L. and *T. vulgaris* L. The seeds (Magic Garden Seeds GmbH^®^) were planted in plastic containers (40 × 20 × 16 cm^3^) containing 700 g of soil. For each treatment, a total of 90 seeds per species (30 seeds × 3 replicates) were sown for each soil type and irrigated with sterile distilled water. Germination was monitored daily for 7 days, considering a seed germinated when the radicle reached a length of ≥2 mm. The experiment was conducted twice. The following germination parameters were derived from the daily records: germination percentage (G), cumulative germination (CG), mean germination time (MGT), and synchrony of the germination process (Z). Moreover, at the end of the germination period, seedling vigor index (SVI), soil stress tolerance index (SSTI), hypocotyl length (HL) and hypocotyl biomass (FW) were determined. The equations used to determine these parameters are provided in Table S3.

### Statistical analysis

The experiment was conducted as a complete randomized design. Data was checked for normality distribution and homoscedasticity within species using the Anderson-Darling and Levene test, respectively. The statistical analysis was performed using ANOVA with the general linear model (GLM) function with three factors (species × substrate × priming treatment) and three replicates. The Tukey’s test was used to determine the statistical differences between factors and treatments, at *p* ≤ 0.05 significance level. All tests were conducted using Minitab statistical software (Minitab LLC, State College, PA, USA).

## Results

### Essential oil composition

A total of 32 volatile compounds were identified in the EO of *L. angustifolia* through GC-MS analysis (Table 1). The major constituents were linalyl acetate (41.01 ± 2.05) and linalool (36.81 ± 1.84%), which together accounted for nearly 78% of the total composition, characterizing the typical aroma profile of lavender oil. Other relevant compounds included limonene (2.34 ± 0.12%), terpinen-1-ol (1.91 ± 0.10), camphor (1.71 ± 0.09), eucalyptol (1.58 ± 0.08), β-myrcene (1.49 ± 0.07), α-terpineol (1.46 ± 0.07) and geranyl acetate (1.11 ± 0.06).

### General effects of treatment

The germination parameters G, SVI and SSTI were significantly influenced by all factors and their interactions, whereas MGT and Z were not significantly affected by soil type (Table 3A). Furthermore, the biometric parameters were influenced by all factors, except for the hypocotyl length (HL), which was not affected by their interactions (Table 3A; Table S2). Moreover, P80 and H_2_O treatments did not differ significantly, consequently H_2_O was used as a unique control.

**Table 3:** Significance of the effects of species, soils, treatments, and their interaction on germination parameters and seedling biometric data. Germination parameters: G, Germination percentage; MGT, Mean germination time; Z, Synchronization index; SVI, Seedling vigor index; SSTI, Soil stress tolerance index (A). Seedling biometric data: F.W., Fresh weight; H.L., Hypocotyl length (B) according to ANOVA analysis. Significance levels are indicated by asterisks: *p* ≤ 0.05 (*), *p* ≤ 0.01 (**), *p* ≤ 0.001 (***), not significant (ns).

A					
ANOVA	G%	MGT	Z	SVI	SSTI
Species (A)	***	***	***	**	***
Treatment (B)	***	***	***	***	***
Soil (C)	***	ns	ns	***	***
A × B	***	**	***	***	***
A × C	***	ns	ns	***	***
B × C	***	ns	*	*	***
A × B × C	***	ns	**	***	***
					
**B**					
**ANOVA**	**F.W.**	**H.L.**			
Specie (A)	***	***			
Treatment (B)	***	***			
Soil (C)	***	*			
A × B	***	***			
A × C	***	ns			
B × C	*	ns			
A × B × C	**	ns			

**Table 4:** Germination percentage G (%), mean germination time (MGT), synchronization index (Z), and seedling vigor index (SVI) of *A. millefolium*, *O. basilicum* and *T. vulgaris* under seed priming with *L. angustifolia* essential oil. Concentrations were 0.1% (EO1), 0.2% (EO2), 0.4% (EO4), 0.8% (EO8) and water (H_2_O) control, across four soils: HM1, HM2 (contaminated), Sal (saline), and C (control). Values are means ± SD. Different letters indicate significant differences within soil (*p* ≤ 0.05).

Species	Soil	Treatment	G	MGT	Z	SVI
*A. millefolium*	HM1	EO1	71.67 ± 6.94^**b**^	3.42 ± 0.30	0.68 ± 0.18	133.61 ± 6.56^**ab**^
		EO2	82.67 ± 4.35^**a**^	3.55 ± 0.29	0.56 ± 0.10	144.92 ± 10.36^**a**^
		EO4	58.89 ± 1.92^**c**^	3.80 ± 0.67	0.56 ± 0.14	99.84 ± 7.25^**c**^
		EO8	47.50 ± 4.19^**d**^	3.38 ± 0.46	0.72 ± 0.27	84.85 ± 8.59^**c**^
		H_2_O	71.67 ± 1.92^**b**^	3.33 ± 0.11	0.66 ± 0.11	128.00 ± 5.55^**b**^
	HM2	EO1	81.11 ± 1.92^**a**^	3.36 ± 0.27	0.73 ± 0.10	152.04 ± 10.93^**ab**^
		EO2	83.33 ± 3.33^**a**^	3.44 ± 0.23	0.70 ± 0.12	155.90 ± 7.26^**a**^
		EO4	80.00 ± 0.00^**a**^	3.60 ± 0.10	0.57 ± 0.04	141.96 ± 5.34^**ab**^
		EO8	71.11 ± 3.85^**b**^	3.37 ± 0.24	0.75 ± 0.13	132.72 ± 7.50^**b**^
		H_2_O	82.50 ± 3.19^**a**^	3.50 ± 0.15	0.62 ± 0.13	147.32 ± 6.53^**ab**^
	Sal	EO1	61.67 ± 2.36^**a**^	4.30 ± 0.20	0.35 ± 0.10	104.8 ± 7.14^**ab**^
		EO2	61.11 ± 1.92^**a**^	3.83 ± 0.28	0.55 ± 0.14	106.64 ± 6.06^**ab**^
		EO4	46.67 ± 3.33^**b**^	3.15 ± 0.08	0.66 ± 0.06	83.50 ± 4.71^**bc**^
		EO8	40.00 ± 6.67^**b**^	4.03 ± 0.64	0.44 ± 0.16	71.19 ± 17.37^**c**^
		H_2_O	71.67 ± 5.77^**a**^	3.66 ± 0.51	0.53 ± 0.12	121.23 ± 8.69^**a**^
	C	EO1	86.67 ± 0.00^a^	4.53 ± 0.09^a^	0.50 ± 0.09	168.93 ± 3.51^a^
		EO2	63.33 ± 3.33^b^	3.66 ± 0.30^bc^	0.57 ± 0.08	105.38 ± 8.45^b^
		EO4	55.56 ± 1.92^c^	3.44 ± 0.08^c^	0.60 ± 0.01	99.91 ± 7.17^b^
		EO8	53.33 ± 2.72^c^	3.34 ± 0.18^c^	0.64 ± 0.07	93.94 ± 12.97^b^
		H_2_O	83.33 ± 2.72^a^	4.09 ± 0.23^ab^	0.41 ± 0.09	150.6 ± 2.20^a^
*O. basilicum*	HM1	EO1	63.33 ± 3.33^**ab**^	2.63 ± 0.37	0.46 ± 0.17	147.12 ± 11.66^**ab**^
		EO2	63.33 ± 9.43^**ab**^	2.72 ± 0.52	0.45 ± 0.12	142.14 ± 23.98^**ab**^
		EO4	57.33 ± 6.41^**b**^	2.76 ± 0.08	0.46 ± 0.10	144.72 ± 16.06^**ab**^
		EO8	55.56 ± 1.92^**b**^	2.26 ± 0.06	0.62 ± 0.05	129.52 ± 7.51^**b**^
		H_2_O	70.00 ± 0.00^**a**^	2.87 ± 0.05	0.39 ± 0.06	170.88 ± 4.52^**a**^
	HM2	EO1	57.78 ± 5.09^**b**^	2.94 ± 0.86	0.49 ± 0.19	143.98 ± 14.67^**ab**^
		EO2	60.00 ± 3.33^**ab**^	3.11 ± 0.68	0.37 ± 0.04	146.40 ± 9.99^**ab**^
		EO4	55.00 ± 1.92^**bc**^	3.39 ± 0.91	0.35 ± 0.04	134.59 ± 3.64^**b**^
		EO8	48.33 ± 4.30^**c**^	3.34 ± 0.27	0.35 ± 0.10	117.62 ± 11.15^**b**^
		H_2_O	67.78 ± 1.92^**a**^	2.99 ± 0.23	0.40 ± 0.13	167.87 ± 19.48^**a**^
	Sal	EO1	46.67 ± 5.77	2.87 ± 0.85	0.48 ± 0.02	108.42 ± 16.01
		EO2	45.56 ± 1.92	3.66 ± 1.14	0.49 ± 0.04	103.9 ± 7.39
		EO4	48.89 ± 5.09	3.51 ± 0.60	0.44 ± 0.04	99.03 ± 10.29
		EO8	41.67 ± 1.92	3.25 ± 0.45	0.39 ± 0.12	90.69 ± 5.27
		H_2_O	46.67 ± 0.00	2.86 ± 0.00	0.38 ± 0.00	99.54 ± 4.95
	C	EO1	56.67 ± 5.77^a^	2.97 ± 0.80	0.42 ± 0.22	142.36 ± 21.12^a^
		EO2	58.89 ± 1.92^a^	3.18 ± 1.20	0.42 ± 0.05	142.21 ± 2.37^a^
		EO4	46.67 ± 3.33^b^	2.94 ± 0.45	0.36 ± 0.09	112.94 ± 8.44^ab^
		EO8	41.11 ± 1.92^b^	3.32 ± 0.01	0.26 ± 0.04	101.89 ± 7.31^b^
		H_2_O	60.00 ± 3.33^a^	2.88 ± 0.25	0.41 ± 0.05	135.66 ± 13.35^a^
*T. vulgaris*	HM1	EO1	68.89 ± 1.92^**ab**^	3.08 ± 0.29	0.38 ± 0.15	131.21 ± 13.85
		EO2	68.89 ± 3.85^**ab**^	3.01 ± 0.20	0.66 ± 0.20	119.08 ± 24.60
		EO4	60.00 ± 6.67^**bc**^	3.33 ± 0.52	0.39 ± 0.15	111.99 ± 22.13
		EO8	56.67 ± 3.33^**c**^	3.01 ± 0.23	0.70 ± 0.09	99.45 ± 9.46
		H_2_O	75.00 ± 1.92^**a**^	3.05 ± 0.09	0.69 ± 0.19	139.03 ± 4.78
	HM2	EO1	71.33 ± 3.80^**b**^	3.73 ± 0.26	0.37 ± 0.13	137.49 ± 16.63^**ab**^
		EO2	80.00 ± 2.72^**a**^	3.20 ± 0.18	0.29 ± 0.06	138.05 ± 13.29^**ab**^
		EO4	63.33 ± 3.33^**c**^	3.53 ± 0.46	0.41 ± 0.07	109.29 ± 9.30^**b**^
		EO8	64.17 ± 3.19^**c**^	3.56 ± 0.78	0.33 ± 0.09	118.82 ± 15.57^**ab**^
		H_2_O	73.33 ± 2.72^**ab**^	3.21 ± 0.40	0.45 ± 0.05	141.30 ± 6.81^**a**^
	Sal	EO1	54.44 ± 5.09^**c**^	3.77 ± 0.77	0.45 ± 0.07^**ab**^	80.14 ± 9.24^**b**^
		EO2	66.67 ± 3.33^**b**^	4.21 ± 1.23	0.34 ± 0.11^**ab**^	92.06 ± 4.14^**ab**^
		EO4	83.33 ± 0.00^**a**^	4.15 ± 0.05	0.29 ± 0.03^**b**^	104.80 ± 1.14^**a**^
		EO8	68.33 ± 7.07^**b**^	3.14 ± 0.77	0.51 ± 0.01^**a**^	103.55 ± 10.60^**ab**^
		H_2_O	83.33 ± 3.33^**a**^	3.90 ± 0.35	0.37 ± 0.07^**ab**^	110.89 ± 11.70^**a**^
	C	EO1	72.22 ± 1.92^**b**^	2.79 ± 0.23	0.49 ± 0.14	150.50 ± 20.43
		EO2	83.33 ± 0.00^**a**^	2.57 ± 0.10	0.54 ± 0.10	174.24 ± 23.08
		EO4	83.33 ± 3.33^**a**^	3.14 ± 0.80	0.50 ± 0.08	157.81 ± 15.75
		EO8	79.33 ± 2.79^**a**^	3.31 ± 0.42	0.38 ± 0.10	165.98 ± 17.88
		H_2_O	70.67 ± 4.35^**b**^	3.32 ± 0.74	0.43 ± 0.13	143.97 ± 16.19

### Germination parameters

Table 4 reported germination percentage (G), mean germination time (MGT), synchronization index (Z), and seedling vigor index (SVI) of the three aromatic species used, under different soils. G was significantly affected by both the EO treatments and the soil types, ranging from 40.00% (Sal-EO8) to 86.67% (C-EO1) (Table 4). The priming treatment on *A. millefolium* revealed a EO dose dependent response based on the soil type. Interestingly, EO2 induced a significant increase (11%) of G in HM1, while in the other substrates the results were comparable to the control. In *O. basilicum* the EO treatment did not improve G in all soil types, revealing a general decrease in Sal. Overall, in both species the EO8 treatment showed a significant reduction of G in all substrates. Finally, in *T. vulgaris* the most interesting results were found in HM2, where the treatment with EO2 significantly increased G compared to the H_2_O control, reaching its maximum of 80.00%, representing the optimum LEO concentration to increase germination. Furthermore, in C the seed primed with EO2-EO8 increased G compared to H_2_O control. The seedling vigor index (SVI) ranged from 84.85 (*A. millefolium*, HM1-EO8) to 168.93 (in *A. millefolium,* C-EO1). *A. millefolium* and *O. basilicum* showed the same G trend with an increase of its value under EO2 in HM1 soil. In addition, all treatments with EO4 and EO8 presented a significant reduction of SVI (Table 3). MGT and Z differed between EO treatments only on *A. millefolium* and *T. vulgaris* of the C and Sal soils. MGT showed a significant highest value under EO1 (4.53) in C, while Z revealed the highest *T. vulgaris* seed germination synchrony under EO8 of the Sal soil (0.51).

### Cumulative germination

Table 5 presents the effects of soil type, treatment and their interaction on cumulative germination (CG). Soil type had a significant impact in all species since day 1. Treatments with LEO significantly affected the CG for *A. millefolium* and *O. basilicum* from day 3 onward but not that in *T. vulgaris*. The interaction between soil and treatment was significant only in *A. millefolium* from day 4 (*p* < 0.01) and for *T. vulgaris* on day 2 (*p* < 0.05), indicating limited combined effects and a species-specific response.

**Table 5:** Results of the ANOVA analysis showing the effects of soil type (A), treatment (B), and their interaction (A × B) on cumulative germination CG (%) over seven days for *A. millefolium*, *O. basilicum*, and *T. vulgaris*. Significance levels are indicated by asterisks: *p* ≤ 0.05 (*), *p* ≤ 0.01 (**), *p* ≤ 0.001 (***), not significant (ns).

Species	Soil and treatment	Significance of cumulative germination (CG)						
		Day 1	Day 2	Day 3	Day 4	Day 5	Day 6	Day 7
*A. millefolium*	Soil (A)	***	***	***	***	***	***	***
	Treatment (B)	ns	ns	***	***	***	***	***
	A × B	ns	ns	ns	**	***	***	***
*O. basilicum*								
	Soil (A)	***	**	***	***	***	***	***
	Treatment (B)	ns	ns	***	***	***	***	***
	A × B	ns	ns	ns	ns	ns	ns	ns
*T. vulgaris*								
	Soil (A)	***	**	***	***	**	**	**
	Treatment (B)	*	ns	ns	ns	ns	ns	ns
	A x B	ns	*	ns	ns	ns	ns	ns

**Figure 1:**
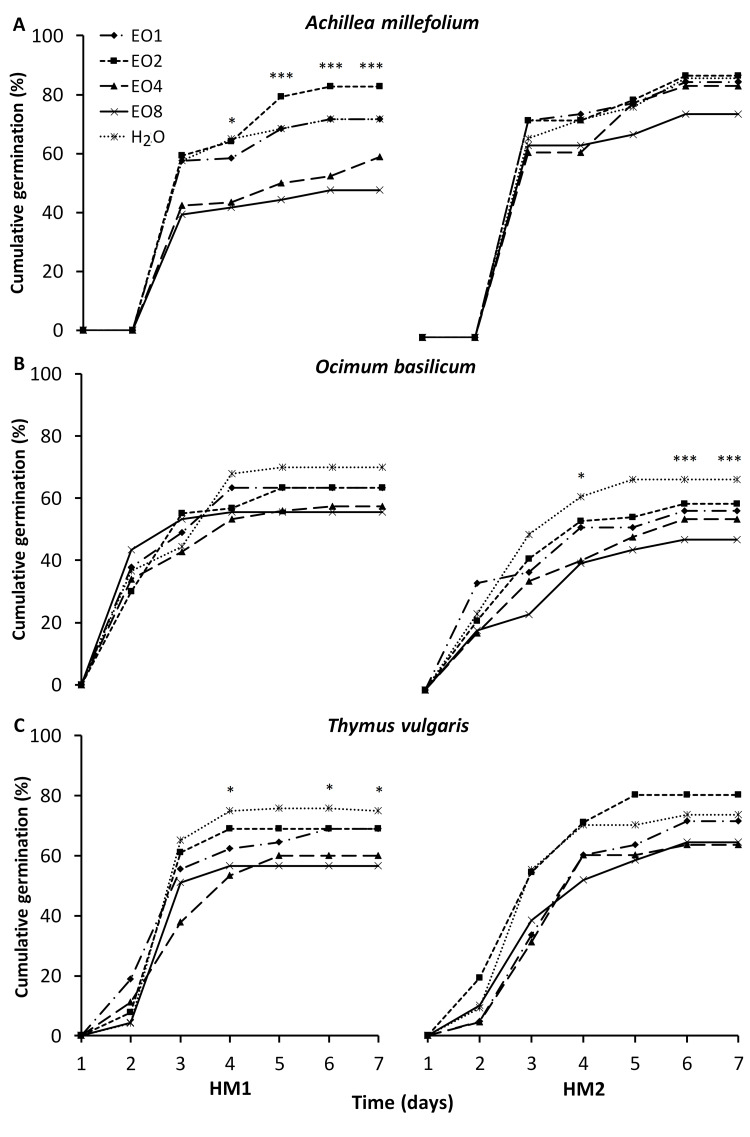
(A–C) Impact of essential oil treatment on cumulative germination of the tested aromatic species in the heavy metal-contaminated soils. Effect of 0.1%; 0.2%; 0.4% and 0.8% concentration of LEO (EO1, EO2, EO4, EO8) in *A. millefolium*, *O. basilicum* and *T. vulgaris* in the heavy metal-contaminated soils (HM1 and HM2) on the cumulative germination percentage. Significance levels are indicated by asterisks: *p* ≤ 0.05 (*), *p* ≤ 0.01 (**), *p* ≤ 0.001 (***) (Tukey test).

**Figure 2:**
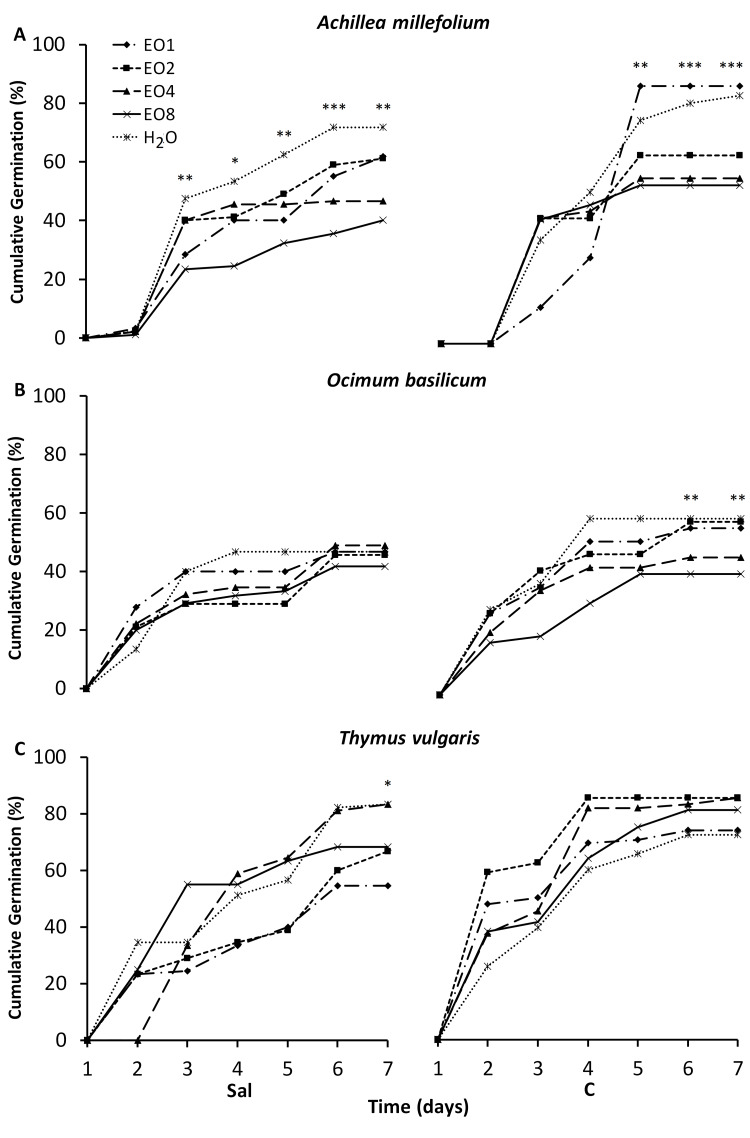
(A–C) Impact of essential oil treatment on cumulative germination of the tested aromatic species in the saline and control soil. Effect of 0.1%; 0.2%; 0.4% and 0.8% concentration of LEO (EO1, EO2, EO4, EO8) in *A. millefolium*, *O. basilicum* and *T. vulgaris* in saline soil (Sal) and control soil (C) on the cumulative germination percentage. Significance levels are indicated by asterisks: *p* ≤ 0.05 (*), *p* ≤ 0.01 (**), *p* ≤ 0.001 (***) (Tukey test).

The CG results are presented in Figs. 1 and 2. *A. millefolium* showed strong variations across treatments and time. In HM1 and Sal soil the EO4 and EO8 treatments had negative effects compared to the other treatments. Interestingly, in HM1 EO2 significantly increased CG from day 3 till the end of the experiment. In C soil, seeds treated with EO1 recorded a significant improvement of CG from day 5 to 7. Similar to the pattern observed in HM1, EO4 and EO8 treatments markedly reduced CG in the control soil. In *O. basilicum,* EO treatments in HM2 and Sal did not improve GC compared to the H_2_O control. Finally, under C soil H_2_O, EO1 and EO2 showed significant difference with EO4 and EO8, which induced a loss of GC. In *T. vulgaris* GC was significantly higher in Sal soil at day 7 under both H_2_O and EO4 treatments. In the HM1 soil, a similar pattern was observed revealing again that H_2_O, as well EO1 and EO2, significantly increased the GC from day 4 to the end of the trial.

### Stress tolerance and biomass

**Figure 3:**
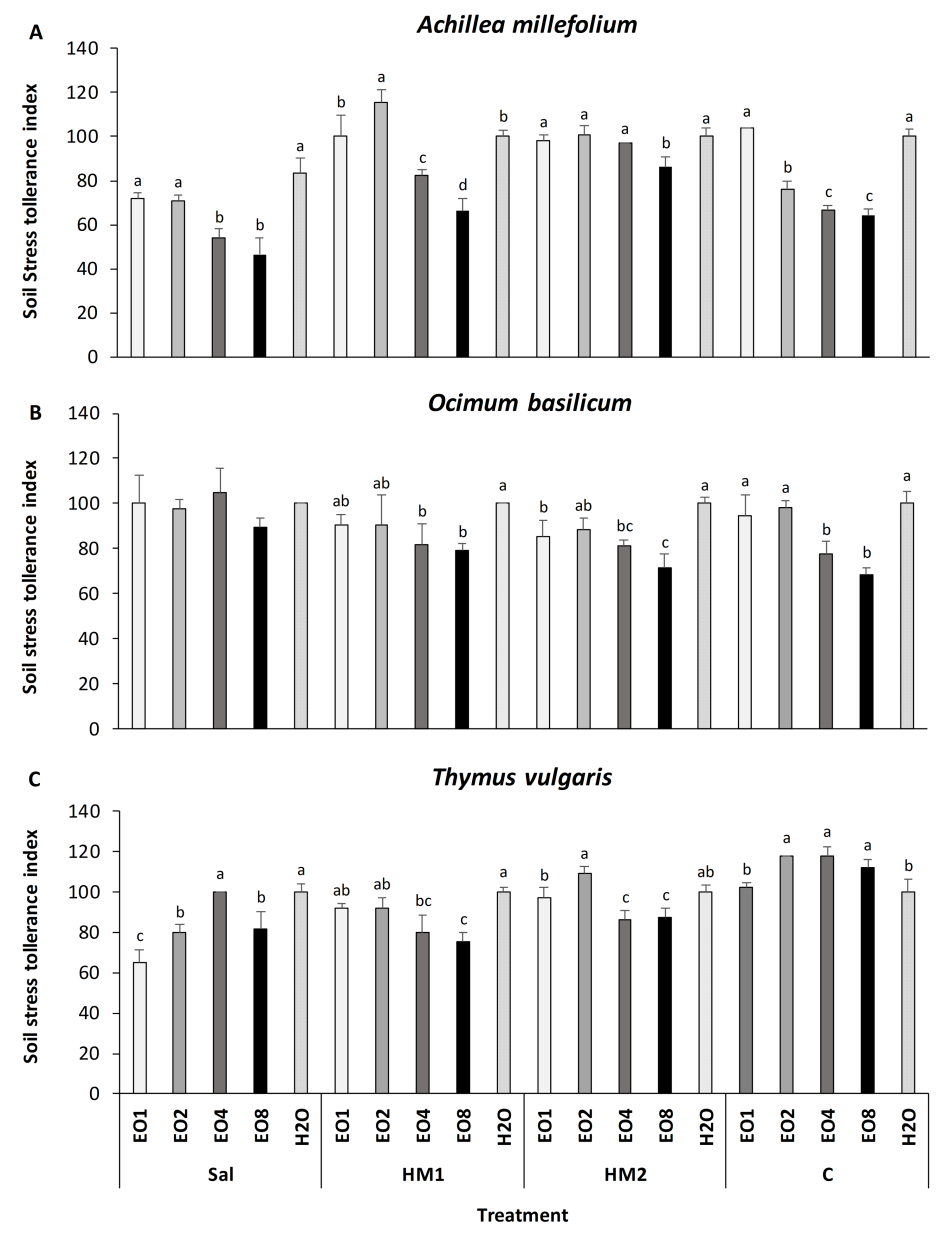
(A–C) Impact of essential oil treatment on soil stress tolerance index of tested aromatic plants in marginal soils. Effect of *L. angustifolia* essential oil at 0.1%, 0.2%, 0.4% and 0.8% concentration (EO1, EO2, EO4 and EO8) and H_2_O in *A. millefolium*, *O. basilicum* and *T. vulgaris*. The experiment was conducted in saline soil (Sal), heavy metal-contaminated soils (HM1 and HM2), and control soil with sand (C), evaluating the Soil Stress Tolerance Index (SSTI). Error bar data shows SD in each treatment. Different letters within the same soil indicate significant differences between treatments (Tukey test, *p*-value ≤0.05).

SSTI revealed clear species-specific responses to priming, with a marked reduction at higher EO concentrations (EO4 and EO8) in *A. millefolium* and *O. basilicum* (Fig. 3; Table 3A). SSTI also significantly varied as a function of the soil type, with the lowest tolerance observed in *A. millefolium* and *T. vulgaris* grown in Sal soil. In *A. millefolium* the EO1 and EO2 priming treatments produced comparable results to the H_2_O control across all soil types. Interestingly, EO2 induced the highest SSTI value, indicating improved performance across treatments and soils. *O. basilicum* EO primed seeds showed results similar to the H_2_O treatment in all soils except HM2, which showed the worst response. In addition, in both *A. millefolium* and *O. basilicum* a significant decrement of SSTI was observed under a high level of EO (EO4 and EO8). A different trend emerged in *T. vulgaris*. Overall, in Sal, HM1, and HM2 soils, EO treatments did not significantly enhance SSTI compared to the H_2_O control. However, under control soil (C), treatments from EO2 to EO8 led to a significant increase in SSTI, with the highest value recorded under EO4.

**Figure 4:**
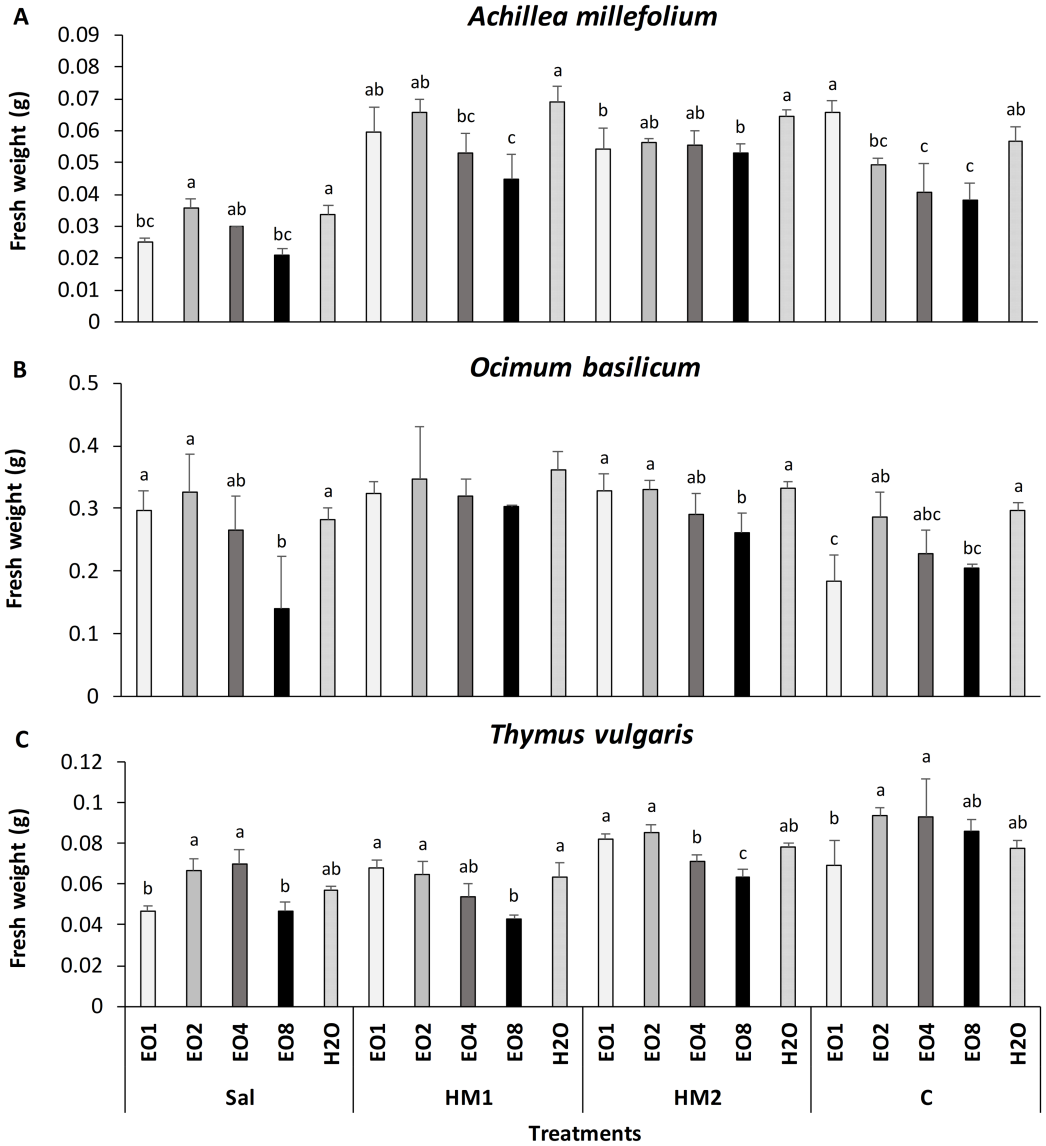
(A–C) Impact of essential oil treatment on biomass of tested aromatic plants in marginal soils. Effect of *L. angustifolia* essential oil at 0.1%, 0.2%, 0.4% and 0.8% concentration (EO1, EO2, EO4 and EO8) and H_2_O in *A. millefolium*, *O. basilicum* and *T. vulgaris*. The experiment was conducted in saline soil (Sal), heavy metal-contaminated soils (HM1 and HM2), and control soil with sand (C), evaluating the fresh weight (F.W) of tested aromatic plants. Error bar data shows SD in each treatment. Different letters within the same soil indicate significant differences between treatments (Tukey test, *p*-value ≤0.05).

*A. millefolium* showed the lowest seedling biomass (FW) under Sal soil, while the highest were observed under HM1 and C (Fig. 4). Across all soils, the EO treatment did not significantly improve the FW and no significant difference was observed compared to control H_2_O priming. In *O. basilicum* EO2 represent the most effective priming treatment in all soil types, although its effects were statistically comparable to H_2_O priming. In addition, in *A. millefolium* and *O. basilicum* the lowest FW was detected in all soil types at EO4 and EO8 with the exception of C, where EO1 had the lowest FW value. For *T. vulgaris*, FW values under EO2 and H_2_O priming were similar across all soils; however, in Sal and C soils, FW increased progressively from EO1 to EO4, followed by a decline at EO8.

## Discussion

In the present study, seed priming with LEO was applied to the seeds of three Mediterranean aromatic plant species seeds, that could be profitably used for the requalification of marginal land such as abandoned mine sites and saline soils. Although seeds are considered tolerant to abiotic stress, this resilience may be only partial in PTE-polluted soils, where germination can be negatively affected (Kranner & Colville, 2011). General toxicity and inhibition of water uptake may significantly reduce germination, and similar inhibitory mechanisms also occur in saline environments due to high electrical conductivity (Munns & Tester, 2008). Seed priming represents a useful strategy to enhance tolerance and improve germination performance under adverse difficult environmental conditions (Oguz et al., 2022; Ben-Jabeur et al., 2019), thereby mitigating the risk of delayed or non-uniform emergence, which can strongly compromise crop establishment and productivity (Jisha, Vijayakumari & Puthur, 2013; Galhaut et al., 2014). Although aromatic plants are generally resilient to abiotic stresses, early developmental stages remain highly sensitive to environmental constraints. Enhancing seed vigor through priming may therefore represent a key agronomic step to improve establishment in salt or contaminated soils, where germination uniformity is often limited.

Among the tested species, *A. millefolium* and *T. vulgaris*, showed an increased G depending on both soil type and priming dose. The dose-dependent response observed in these two species is consistent with the findings of Oğuz, Oğuz & Güler (2023) in wheat seeds coated with a mixture of aromatic plant EOs (including lavender). Those authors reported a significant increase of G at 0.05% EO, followed by a progressive decrease at 0.10% and 0.25%, suggesting a narrow optimal concentration range. This pattern aligns with our results, where seed priming with LEO at 0.1% and 0.2% led to the highest G, while higher concentrations (0.4% and 0.8%) resulted in reduced germination. However, this stimulatory effect was not consistent across all species or soils. In several cases, germination under LEO priming was comparable to, or even lower than the water control, indicating the biostimulant response species- and soil-specific. To the best of our knowledge, EO seed priming treatment with LEO has not been previously tested in aromatic species in order to improve their germination performance under abiotic stress; prior EO applications on aromatic crops mainly addressed seed sanitation or allelopathic and bioherbicidal effects rather than priming sensu stricto (Konstantinović et al., 2022; Rahimi et al., 2013; Terzić et al., 2023). Overall, these findings suggest that EO-based treatments exhibit a threshold-dependent effect, where lower doses stimulate germination, whereas higher doses may exert phytotoxic effects. Galhaut et al. (2014) also reported that priming treatments with gibberellic acid, in presence of Cu and Zn as soil contaminants, induce an increase of G with the increasing of the treatment time reaching the maximum of G at 18 h and 12 h respectively, and after that, a further decrease of germination. These results also support the idea that seed priming could increase G by stimulating the production of a wide range of temporary signals *i.e.,* reactive oxygen species (ROS) and its scavenging antioxidant metabolites (Maffei, Mithöfer & Boland, 2007). Although the involvement of ROS and hormone-related signaling pathways is plausible, this interpretation remains hypothetical since these parameters were not measured in the present study. This mechanism could be applied to several abiotic stress inductors associated with soil (excessive salt as well as chemical pollutants). Lutts et al. (2016) suggested that this “stress activator mechanism” induced a sort of stress memory in seeds that is responsible for a more efficient adaptation to subsequent episodes of abiotic stress. We cannot exclude that this type of “stress memory” involves all the early-stage development of seeds, including the seedling development. These considerations are further supported by our experimental data, particularly for *A. millefolium*, which showed increased SVI and FW under EO priming treatments, especially at 0.1–0.2% concentrations (Table 4 and Fig. 4). As expected, each plant species responded differently to soil and priming treatment. In our study, *O. basilicum* showed a limited response in terms of G and SVI to the priming treatment compared to H_2_O (control). Specifically, in basil grown in the HM2 substrate, a significant decrease of G was observed under EO1 compared to H_2_O and no significant increase of G or SVI in any of the tested treatment and soils was found. This highlights a possible negative effect of priming in this species, soil, and their interaction (Table 4).

Soil type and LEO treatment significantly affected CG of *A. millefolium*, *O. basilicum* and *T. vulgaris*, with species-specific and dose-dependent responses. Rahimi, Bidarnamani & Shabanipoor (2015), investigated the effect of different concentrations of *Lavandula vera* DC. EOs (0.025%, 0.050%, 0.075%, and 0.1%) on *Cynodon dactylon* L. Pers. weeds, observed a concentration-dependent reduction in germination rate. In our study, the general reduction of CG in all species observed at 0.4% and 0.8% doses can be explained by the presence of oxygenated monoterpenes and other compounds (linalool, linalyl acetate, limonene) known as high inhibitors of seed germination and seedling growth (Singh et al., 2002; Abrahim et al., 2000). Moreover, Ibáñez & Blázquez (2019) reported that *L. angustifolia* EO affects seed germination and development of *L. multiflorum* Lam., *Echinochloa crus-galli* (L.) Beauv. and *Nicotiana glauca* Graham for its herbicidal proprieties. However, in *A. millefolium* and *O. basilicum* at HM1 or C and HM2 or C soils respectively, doses between 0.1% and 0.2% had no repressive effect on germination performance. In fact, although not significant, in some cases germination was higher. In fact, low levels of ROS and their controlled accumulation positively affects germination, as it allows for increased activation of genes involved in water uptake (Oğuz, Oğuz & Güler, 2023).

Improving the abiotic stress tolerance at the seed germination stage represents an important agronomic trait that increases crop productivity and could be extremely useful in the recovery of marginal, degraded, and abandoned areas. Our results indicate that species priming treatment as well as the soil type significantly affect the soil tolerance index of the plants. The priming treatment indicated an increase of tolerance in *A. millefolium* and *T. vulgaris* in the presence of HM1 and HM2 soils with ∼0.2% significant increase of tolerance to heavy metals. In our case, these two aromatic species showed (in the presence of the EO priming) different tolerance indexes. Similarly, Kranner & Colville (2011) reported that *Arabidospis thaliana* (L.) Heynh., *Bowiea volubilis* Harv. ex Hook.f.*, Eucomis autumnalis* (Mill.) Chitt*., Merwilla natalensis* (Planch.) Speta and *Eruca sativa* Mill., have a different tolerance to a wide range of soil metals and contaminants. In our data the EO2 treatment induced an increase of tolerance in HM1 and HM2 soils compared to the control (H_2_O) in *A. millefolium* and *T. vulgaris*. Under stress conditions due to heavy metals or salinity, the production of ROS is probably induced, causing the activation of oxidative stress pathways that include molecular, biochemical and morphological responses. High level of ROS can cause many physiological alterations including the germination and seedling development (Oğuz, Oğuz & Güler, 2023). The seed germination is highly regulated by ROS. Indeed, ROS activate gene expression and hormone production (*i.e.,* auxin, gibberellin, ethylene) that are responsible for the chain of events that induce germination activation (Bailly & Merendino, 2021). If ROS are too high, the oxidative damage occurs in cells causing an inhibition of germination; conversely, low ROS level has a positive effect in seed germination (Bailly, 2019; Kurek, Plitta-Michalak & Ratajczak, 2019). As reported by Oguz et al. (2022), phenolic compounds, plant extracts and EOs can have a biostimulant effect on seed, root and shoot. EOs are rich in organic compounds that have shown antioxidant activity protecting plant tissue from oxidative stress and hormone degradation (auxin) (Amorati, Foti & Valgimigli, 2013; De Klerk et al., 2011). Basically, the organic compounds in the EO protect the plant from oxidative stress, keeping the ROS at a low level and reducing hormone unbalance, improving the plant’s physiological activities such as germination and seedling development (De Klerk et al., 2011). Finally, we observed that under C substrate, only *T. vulgaris* showed a significant increase in Seedling Stress Tolerance Index at 0.2%, 0.4%, and 0.8% treatments compared to the untreated seeds (H_2_O). These results are likely related to the general low level of ROS and oxidative state associated with the priming solutions. Although sand itself does not represent a source of stress, however, the presence of LEO induced a positive effect. In this case, the organic compounds present in LEO can positively interact with the endogenous hormones, balancing their content and preventing auxin degradation (Oğuz, Oğuz & Güler, 2023). All these events induce an increase in germination and tolerance index.

The integration of LEO priming at low concentration (0.1–0.2%) improved germination performance, early seedling development and reduced seedling stress, in some species and soils; while higher doses showed inhibitory effects. This highlights a dose, soil, and species-dependent biostimulant potential rather than a consistent enhancement. This combined approach, on the same contaminated soils, strengthens the potential of already tolerant aromatic species by boosting their resilience under stress conditions with respect to unprimed seeds (Piga et al., 2025). These findings support a sustainable strategy for recovery and valorization of marginal lands.

## Conclusions

This study showed that seed priming with lavender essential oil (LEO) affects germination, growth and stress tolerance in three aromatic plants (*A. millefolium*, *O. basilicum*, and *T. vulgaris)*, with species- and dose-dependent responses. Trials were conducted on four Sardinian soils: two potentially toxic elements (PTEs) polluted (HM1, HM2), one saline (Sal), and a sandy control (C). Low EO concentrations enhanced germination percentage (G), seedling vigor index (SVI), and fresh weight (FW) particularly in *A. millefolium* and *T. vulgaris* even in the presence of heavy metals and salinity, suggesting species-specific resilience and adaptability. Compared to the water control, EO priming at low concentrations also improved cumulative germination in *A. millefolium* and *O. basilicum*, leading to earlier and faster seedling emergence. In contrast, higher doses (≥0.4%) delayed or reduced cumulative germination compared to the water control. However, *T. vulgaris* maintained comparable or slightly improved performance across treatments. The Soil Stress Tolerance Index confirmed that EO priming—especially at 0.2%—increased the stress tolerance to PTEs-polluted soils in *A. millefolium* and *T. vulgaris*, while limited responsiveness to LEO was observed in *O. basilicum* across all soil types, suggesting its lower suitability in degraded environments. These results confirmed the potential of LEO as a natural priming agent to enhance seedling establishment under abiotic stresses induced by saline and PTEs soils, with potential applications to support their recovery through the cultivation of aromatic species for EO extraction. Further studies under open field conditions are needed to validate these findings and assess their effectiveness in land reclamation efforts.

## Supplemental Information

10.7717/peerj.20717/supp-1 Supplemental Information 1 Supplementary material on the materials and methods LEO dilutions used in the experiments, biometric data and Formulas to calculate germination related variables.

10.7717/peerj.20717/supp-2 Supplemental Information 2 Raw germination data of the aromatic species

## Additional Information and Declarations

### Data Availability

The following information was supplied regarding data availability: The raw data is available in the Supplemental File.
