# Supplementary material for: Seed priming with lavender essential oil increases germination and seedling growth in saline and potentially toxic elements-contaminated soils
Source: PeerJ. 2026 Apr 8;14:e20717. doi: 10.7717/peerj.20717 (PMC13069936; doi:10.7717/peerj.20717)
Supplement: Supplemental Information 1 — LEO dilutions used in the experiments, biometric data and Formulas to calculate germination related variables. [file peerj-14-20717-s001.pdf]

SUPPLEMENTARY MATERIAL

Supplementary Table 1. Description of the solution used for seed priming. The percentage of *L. angustifolia* essential oil (EO), H_2O and Polisorbate 80 (P80) and the related code is also reported.

Code	EO (%)	Treatment	Final Volume (mL)
LEO4%	4%	1.2 mL LEO + 0.18 mL P80 + 28.62 mL H_2O	30
H_2O	0.0%	10 mL H_2O	10
P80	0.0%	0,001 mL P80 + 9,999 mL H_2O	10
EO1	0.1%	0.25 mL LEO4% + 9.75 mL H_2O	10
EO2	0.2%	0.50 mL LEO4% + 9.50 mL H_2O	10
EO4	0.4%	1.00 mL LEO4% + 9.00 mL H_2O	10
EO8	0.8%	2.00 mL LEO4% + 8.00 mL H_2O	10

Supplementary Table 2. Mean hypocotyl length (cm) of *A. millefolium*, *O. basilicum*, and *T. vulgaris*. The following substrate were considered: Arborea (Sal); Montevecchio (HM1); Su Suergiu (HM2) and Sand as control (C). The treatment tested were 0,1; 0,2; 0,4 and 0,8 % concentration (EO1; EO2; EO4 and EO8) of *L. angustifolia* EO and only water (H_2O). Values are presented as mean ± standard deviation.

Soil	Treatment	Hypocotyl lenght (cm)		
		A. millefolium	*O. basilicum*	*T. vulgaris*
Sal	EO1	1.73±0.25	2.33±0.22	1.49±0.18^{ab}
	EO2	1.75±0.24	2.31±0.33	1.39±0.15^{ab}
	EO4	1.79±0.20	2.03±0.36	1.26±0.14^{b}
	EO8	1.73±0.24	2.16±0.34	1.52±0.18^{a}
	H_2O	1.70±0.18	2.14±0.37	1.35±0.22^{ab}
HM1	EO1	1.86±0.27	2.38±0.35	1.98±0.34
	EO2	1.76±0.26	2.24±0.24	1.81±0.36
	EO4	1.69±0.28	2.53±0.32	1.88±0.31
	EO8	1.79±0.27	2.36±0.27	1.75±0.20
	H_2O	1.79±0.21	2.47±0.27	1.87±0.29
HM2	EO1	1.85±0.25	2.49±0.28	1.93±0.34
	EO2	1.87±0.27	2.43±0.30	1.76±0.32
	EO4	1.77±0.23	2.44±0.29	1.73±0.26
	EO8	1.87±0.30	2.45±0.29	1.87±0.28
	H_2O	1.78±0.27	2.43±0.34	1.93±0.24
C	EO1	1.95±0.26^{a}	2.51±0.21	2.08±0.28
	EO2	1.66±0.23^{b}	2.41±0.30	2.09±0.39
	EO4	1.80±0.29^{ab}	2.42±0.29	1.86±0.30
	EO8	1.74±0.28^{ab}	2.48±0.30	2.06±0.26
	H_2O	1.81±0.25^{ab}	2.26±0.24	2.01±0.27

Supplementary Table 3. Formulas to calculate germination related variables (G: Germination percentage; MGT: Mean germination time; Z: Synchrony of the germination process; SVI: Seedling vigor index; Soil stress tolerance index: SSTI).

Variable	Formula	Explanation
G	$\frac{\sum_{i=1}^{k} n_i}{N} \times 100$	n_i = number of seeds germinated at interval i<br>N = total number of seeds germinated.
MGT	$\frac{\sum_{i=1}^{k} n_i t_i}{\sum_{i=1}^{k} n_i\, n_i}$	$n_i t_i$ = the product of seeds germinated at interval i with the corresponding time interval.
Z	$\frac{\sum_{i=1}^{k} C_{ni,2}}{C_{\sum_{i=1}^{k} ni,2}}$	$Cn_i.2$ = Combination of the seeds germinated in the ith time. two by two. thus, ranging between 0 (when at least two seeds could germinate. one at each time) and 1 (when germination of all seeds occurs at the same time).
SVI	HL x G%	HL correspond to hypocotyls length.<br>G% correspond to germination percentage.
SSTI	$\frac{G_{Treatment}}{G_{H_2O}} \times 100$	$G\%_{Treatment}$ = germination percentage for each treatment and soil (dose 0.01, 0,02, 0.04, and 0.08 percent).<br>$G\%H_2O$ = mean germination percentage for the dose 0% for each soil type.
